# New Flurbiprofen Derivatives: Synthesis, Membrane Affinity and Evaluation of *in Vitro* Effect on β-Amyloid Levels

**DOI:** 10.3390/molecules180910747

**Published:** 2013-09-03

**Authors:** Piera Sozio, Lisa Marinelli, Ivana Cacciatore, Antonella Fontana, Hasan Türkez, Gianfabio Giorgioni, Dario Ambrosini, Francesco Barbato, Lucia Grumetto, Stephanie Pacella, Amelia Cataldi, Antonio Di Stefano

**Affiliations:** 1 Department of Pharmacy, “G. D’Annunzio” University, Via dei Vestini 31, Chieti 66100, Italy; 2 Department of Molecular Biology and Genetics, Erzurum Technical University, Erzurum 25240, Turkey; 3 Medicinal Chemistry Unit, School of Pharmacy, University of Camerino, via S. Agostino 1, Camerino 62032, Italy; 4 Center for Drug Discovery 116 MU, Northeastern University, 360 Huntington Avenue, Boston 02115-5000, MA, USA; 5 Department of Pharmacy, Università di Napoli Federico II, Via D. Montesano 49, Naples 80131, Italy; 6 Department of Medicine and Ageing Sciences, “G. D’Annunzio” University, Via dei Vestini 31, Chieti 66100, Italy

**Keywords:** Alzheimer’s disease, beta amyloid peptide, flurbiprofen, γ-secretase

## Abstract

Alzheimer’s disease (AD) is characterized by irreversible and progressive loss of memory and cognition and profound neuronal loss. Current therapeutic strategies for the treatment of AD have been directed to a variety of targets with the aim of reversing or preventing the disease but, unfortunately, the available treatments often produce no significant clinical benefits. During the last decades compounds that inhibit or modulate γ-secretase, reducing β amyloid (Aβ) levels, have been considered as potential therapeutics for AD. Among these the (*R*)-enantiomer of flurbiprofen (FLU) seems to be very promising, but it shows low brain penetration. In this study, in order to improve the properties of FLU against Alzheimer’s pathogenesis we synthesized some novel FLU lipophilic analogues. Lipophilicity of the new molecules has been characterized in terms of *c*log*P*, log K_C18/W_ and log K _IAM/W_ values. Permeability has been determined in both gastrointestinal PAMPA (PAMPA-GI) at different pH values and in brain blood barrier PAMPA (PAMPA-BBB) models. They were also tested for their ability to inhibit *in vitro* γ-secretase activity using rat CTXTNA2 astrocytes. Interestingly, the investigated molecules demonstrated to reduce Aβ 42 levels without affecting the amyloid precursor protein APP level in a clear concentrations-dependent manner.

## 1. Introduction

Alzheimer’s disease (AD) is one of the most common types of dementia, characterized by irreversible and progressive loss of memory and cognition and profound neuronal loss [1]. Current therapeutic strategies for the treatment of AD have been directed to a variety of targets with the aim of reversing or preventing the disease but, unfortunately, the available treatments produce limited clinical benefits [2]. During the last decades many investigations have been directed to the modulation of amyloid precursor protein (APP) transcription, including α-, β-, and γ-secretase. In particular, compounds that inhibit or modulate γ-secretase can be considered as a potential therapeutics for AD because their use appears to be a rational strategy to prevent senile plaque (SP) formations [3].

**Figure 1:**
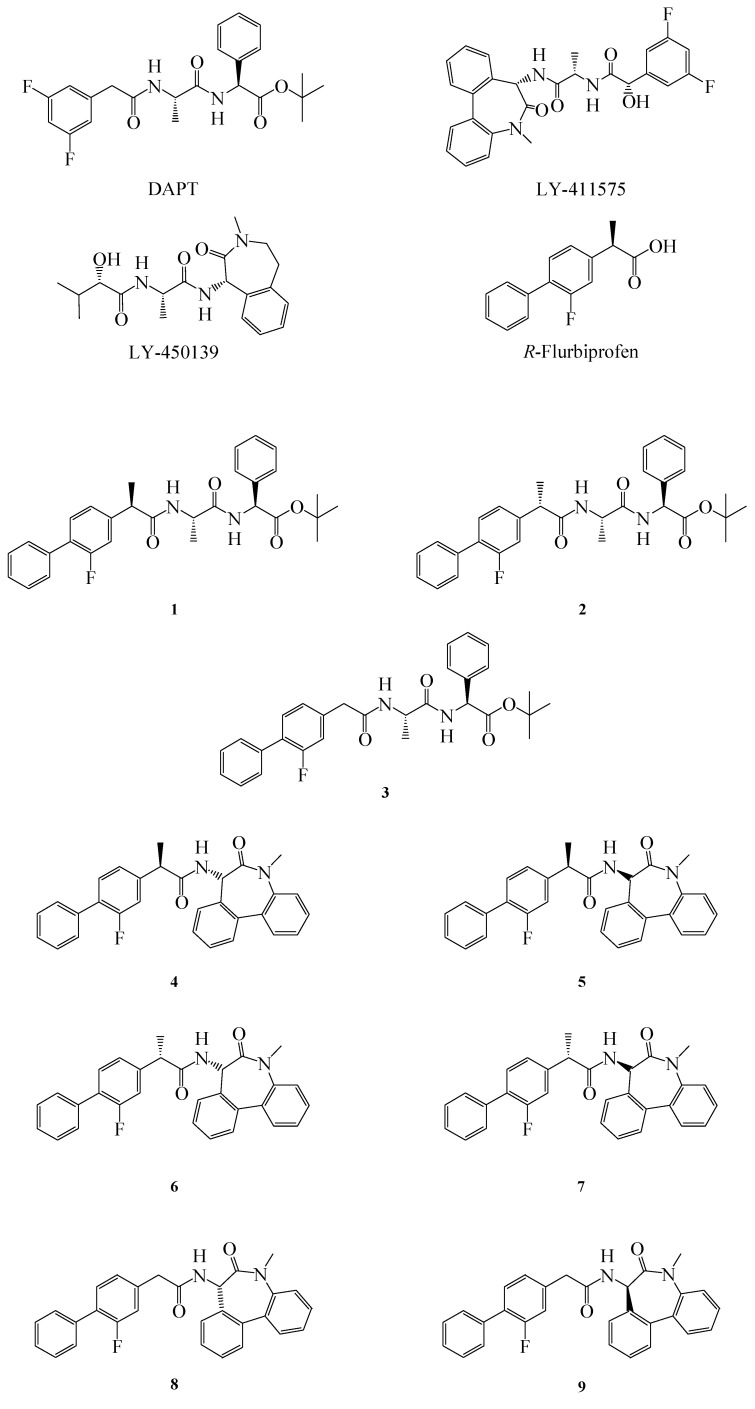
Structure of GSIs, GSMs and compounds **1**–**9**.

The first γ-secretase inhibitor (GSI) with *in vivo* efficacy was DAPT (Elan and Eli Lilly), followed by LY-450139 (also known as semagecestat) and LY-411575 (Eli Lilly) (Figure 1). When administered in mice they inhibit APP cleavage, thus dose dependently reducing β amyloid (Aβ) levels and avoiding plaque formation [4].The major problem associated with these inhibitors is their non selective targeting towards APP. As a matter of fact they also inhibit the cleavage of the Notch receptor, causing embryological defects, interferences in B and T lymphocytes proliferation, and abnormalities in the gastrointestinal (GI) tract [5,6].

These toxic effects can be avoided by substituing GSI with γ-secretase modulators (GSMs). Indeed, γ-secretase is a multi-subunit protease complex constituted by at least four components (presenilin, nicastrin, aph-1 and pen-2). Two aspartates moieties lying at the interface of presenilin constitute the catalytic site [7].

γ-Secretase can cut APP at different positions along its chain (referred to as γ, ε, and ζ), but Aβ is released only after a γ-cut generating fragments whose length ranges from 37 to 43 amino acid residues. The prevalence of one Aβ type rather than another influences its pathogenicity, because only the Aβ 42 is prone to aggregate and thus to form plaques. The GSIs, by targeting the catalytic pocket, inhibit the production of all Aβ peptides without differentiating the processing of APP or Notch. On the contrary, the GSMs can act either by blocking the Aβ production (Notch-sparing GSIs) or by modulating the ratio between long and short Aβ peptides (GSMs, properly named), without changing the total amount of Aβ produced. In both case GSMs do not affect Notch signaling [6]. Among the GSMs, some non-steroidal anti-inflammatory drugs (NSAIDs), like flurbiprofen, ibuprofen, and indomethacin, seem very promising. They are capable of reducing oligomer formation by allosteric modulation of presenilin and their activity as GSMs is not correlated with their anti-cyclooxygenase activity [7,8].

The most studied NSAIDs in this context is the (*R*)-enantiomer of flurbiprofen (FLU) (Figure 1), but unfortunately, results from the Myriad Flurizan trials have attributed to FLU poor potency due to very poor brain penetration [9]. Therefore, new derivatives with better pharmacokinetic and pharmacodynamic properties are required [10]. In order to improve the properties of FLU, we synthesized some novel FLU lipophilic analogues and tested their potency on Aβ level reduction. Starting from a previous work of Peretto and coworkers on the structure-activity relationships (SAR) for FLU analogues we designed new peptide derivatives of FLU (compounds **1**–**9**, Figure 1), in which the carboxylic group was replaced by an amide moiety and linked to L-alanine-L-phenylglycine-*t*-butylester or L*-*alanine-benzodiazepine (N1-[(7*S*)-5-methyl-6-oxo-6,7-dihydro-5*H*-dibenzo[b,d]azepin-7-yl]-L*-*alanine) residues, in order to mimic the structure of the effective inhibitors DAPT and LY-411575, respectively [8]. The substitution in the α position was also considered, due to the different activity of the (*R*)- and (*S*)- enantiomers of FLU on both COX-1 and Aβ42 [11].

The pharmaceutical profile of the new agents was studied in order to determine the potential of both their intestinal and Blood Brain Barrier (BBB) permeability. Therefore, the present study included evaluation of solubility in Fasted State Simulated Intestinal Fluid (FASSIF), *n*-octanol partition coefficient, membrane phospholipid affinity by immobilized artificial membrane (IAM) chromatography and permeability coefficient by the Parallel Artificial Membrane Permeability Assay (PAMPA) and *in vitro* activity [12].

## 2. Results and Discussion

**Scheme 1:**
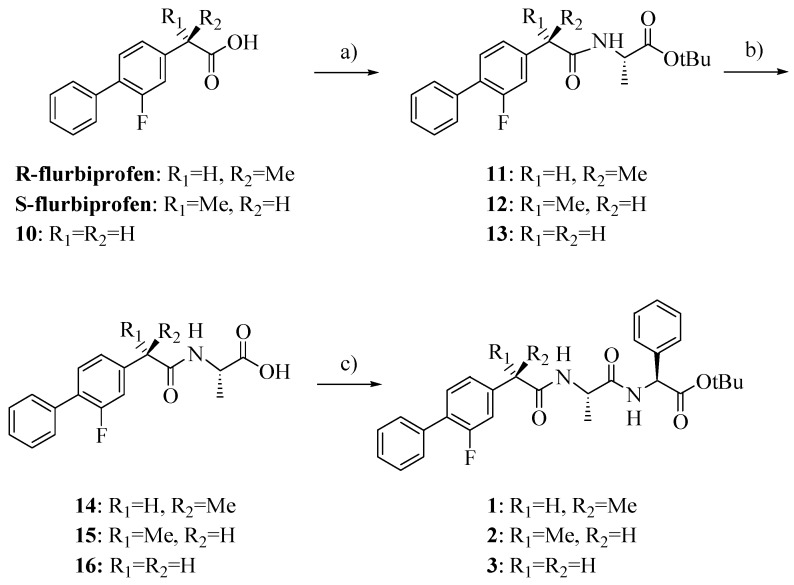
Synthesis of compounds **1**–**3**.

**Scheme 2:**
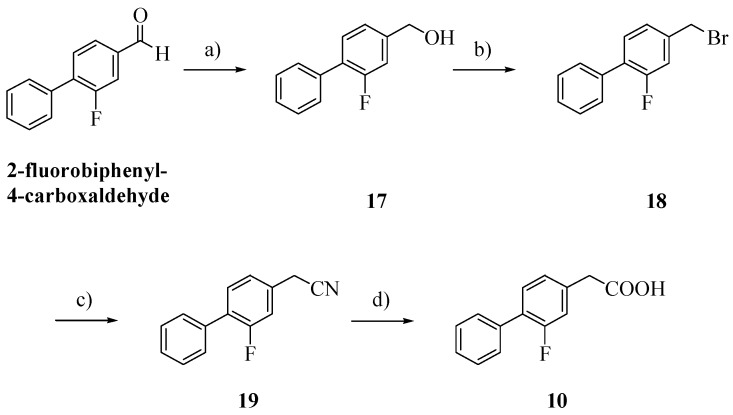
Synthesis of FLU derivative **10**.

Compounds **1**–**3** were synthesized as outlined in Scheme 1 by using stepwise elongation of the peptide chain in the C-to-N direction via standard solution phase procedures. Amides **14**–**16** were prepared by conventional DCC/HOBt condensation of FLU or its derivative **10** with L-alanine-*t-*butylester hydrochloride and subsequent acidolysis with TFA; coupling with L*-*phenylglycine-*t-*butylester hydrochloride gave the final products **1**–**3** [13,14]. FLU derivative **10** was prepared as previously reported with a few slight modifications and in higher yield (Scheme 2) [15].

*Reagents*: **a**) L*-*alanine-*t-*butylester hydrochloride, HOBt, Et_3_N, DCC, DCM; **b**) TFA; **c**) L*-*phenylglycine-*t-*butylester hydrochloride, HOBt, Et_3_N, DCC, DCM.

*Reagents*: **a**) dry MeOH, NaBH_4_, THF; **b**) 48% hydrogen bromide; **c**) KCN, EtOH, H_2_O; **d**) NaOH, EtOH, H_2_O.

Alcohol **17**, obtained by reduction of 2-fluorobiphenyl-4-carboxaldehyde with NaBH_4_, was treated with 48% hydrogen bromide and then with potassium cyanide [16,17]. Hydrolysis of the cyano group afforded the biphenylacetic acid **10** [8].

**Scheme 3:**
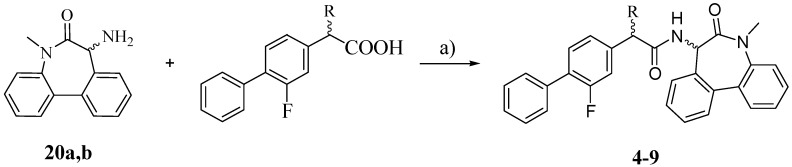
Synthesis of compounds **4**–**9**.

The synthesis of the target compounds **4**–**9** was carried out as outlined in Scheme 3: the proper carboxylic acid (R-FLU, S-FLU or the unsubstituted FLU derivative **10**, respectively) was coupled with pure (*S*)- and (*R*)-5-amino-7-methyl-5,7-dihydro-6*H*-dibenz-[b,d]azepin-6-one (**20a** and **20b**) in the presence of *N*-(3-dimethylaminopropyl)-*N′*-ethylcarbodiimide hydrochloride (EDCI) and HOBt [18].

*Reagents*: **a**) EDCI, HOBt, THF.

**Scheme 4:**
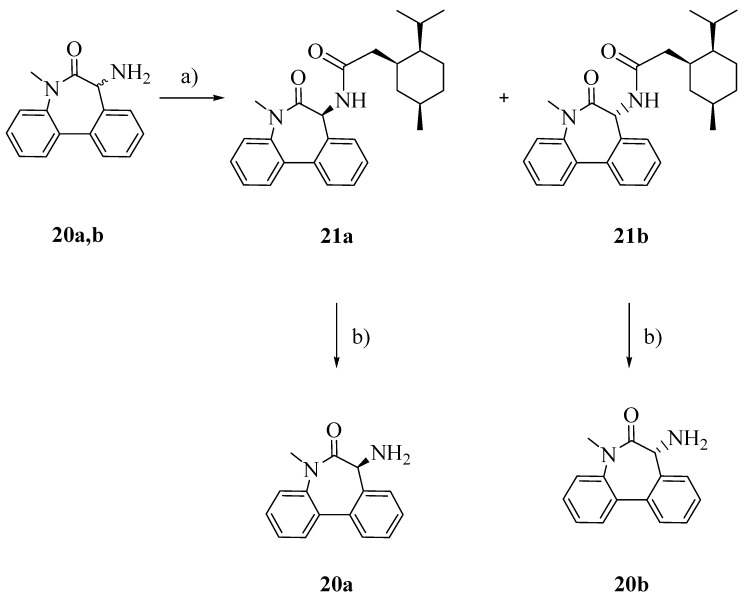
Separation of enentiomers **20a** and **20b**.

To obtain the pure enantiomers **20a** and **20b**, racemic aminolactam **20** was conjugated with (1*S*)-(+)-menthyl chloroformate (Scheme 4), separated by flash column chromatography and finally hydrolyzed in the presence of triflic acid and trifluoroacetic acid [19].

*Reagents*: **a**) TEA, (1S)-(+)-menthyl chloroformate, Et_2_O; **b**) CF_3_COOH, CF_3_SO_3_H, CH_2_Cl_2_.

**Scheme 5:**
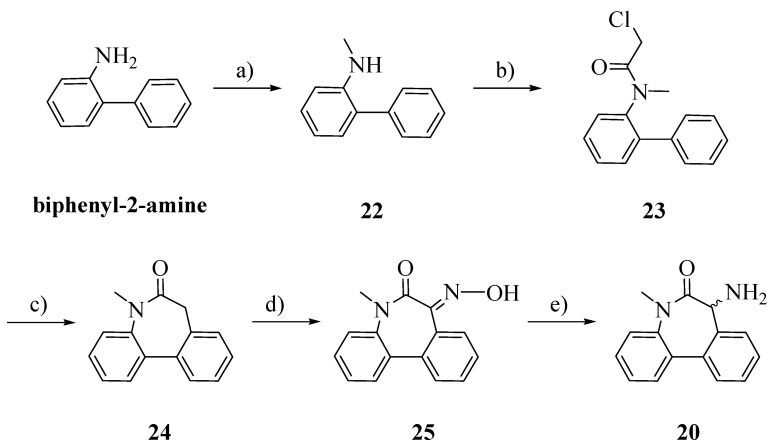
Synthesis of racemic aminolactam **20**.

The racemic aminolactam **20** was prepared as previously reported, with slight modifications (Scheme 5) [20]. Starting from biphenyl-2-amine, an amination/reduction reaction with formaldehyde and palladium on activated charcoal gave the methylated product **22** [21]. Compound **23** was obtained by acetylation of **22** with chloroacetyl chloride. The cyclization, perfomed by a Friedel-Craft’s intramolecular alkylation, allowed us to obtain **24** [22]. The oxime **25** was obtained by treating **26** with potassium bis(trimethylsilyl)amide (KHMDS) and isopentyl nitrite. Reduction in a Parr autoclave with palladium on activated charcoal afforded **20** [23].

*Reagents and Conditions*: **a**) HCOH, NH_4_OCOH, Pd/C, iPrOH, H_2_O; **b**) ClCOCH_2_Cl, CH_2_Cl_2_, DIPEA; **c**) AlCl_3_, 100 °C–170 °C; **d**) isopentyl nitrite, KHMDS, Ph-CH_3_; **e**) H_2_, Pd/C, HCl, EtOH.

The pharmaceutical profile of the new agents was studied in order to determine the potential of both their intestinal and BBB permeability. Besides, waters solubility of the investigated compounds was determined for establishing the GI dissolution rate. Before partitioning into GI wall, a solid dosage form should solubilize in the gastric fluids. For a good absorption profile from the GI lumen, a drug should have solubility higher than 100 μg/mL [24]. On the other hand, the studied compounds demonstrated to have low water and FaSSIF solubility (lower than 100 ng/mL and 10 μg/mL, respectively).

Nevertheless, this drawback could be overcome by their high *n*-octanol/water partition coefficients. Indeed the synthesized molecules, designed by replacing the FLU ionizable carboxylic function with lipophilic unionizable substituents, are characterized by high *n*-octanol/water lipophilicity values, as confirmed by their *c*log*P* values, that are higher than those of (*R*)-flurbirpofen and other γ-secretase inhibitors (Table 1).

**Table 1:** *c*log*P* of compounds **1**–**9**.

1-2	3	4-7	8-9	R-FLU	DAPT	LY-411575	LY-450139	Nisoldipine	Lacidipine
5.71	5.40	6.08	5.77	3.81	3.45	3.57	1.02	4.53 ^a^	5.56 ^a^

^a^ Experimentally determined values.

Similarly, due to their high lipophilicity, experimental *n*-octanol/water lipophilicity values of enantiomers **8**/**9** were determined by RP-HPLC because their characterization through the “classical” shake-flask method was expected to be difficult and scarcely reproducible [25]. The retention factor, determined in acetonitrile/water mixtures and extrapolated to 100% water (K_C18/W_) for enantiomers **8**/**9** was 3.94 (data not shown).

By comparing this value with K_C18/W_ values of two analogously non-ionizable compounds such as nisoldipine (*i.e.* K_C18/W_ 2.99) and lacidipine (*i.e.* K_C18/W_ 4.01) and considering their corresponding log*P* values (*i.e.* 4.53 and 5.56, respectively), a log*P* value of 5.49 for enantiomers **8**/**9** can be calculated. This latter value is strictly comparable with the *c*log*P* reported in Table 1 [26].

With the aim to evaluate a parameter that closely ensures the affinity of our neutral compounds for membrane phospholipids, we chose to measure their retention factors on phospholipid-based stationary phase by performing IAM-HPLC measurements (Table 2). Indeed, it is well known that phospholipid layers, being an anisotropic partition phase, have superior biomimetic properties than n-octanol for neutral compounds [27].

**Table 2:** Log*K_IAM/W_* of compounds **1**–**9**.

1	2	3	4/7	5/6	8/9	R-FLU	DAPT	LY-411575	LY-450139	Nisoldipine	Lacidipine
3.67	3.80	3.64	3.21	3.93	3.58	1.82	1.61	1.85	0.11	2.63	3.52

It is worth noting that, although the experimental measurements were performed on all the compounds under investigation, being the IAM phase not enantioselective, a single log*K_IAM/W_* value for each of the three enantiomer pairs, *i.e.*, compounds **4** and **7**, **5** and **6**, **8** and **9**, is reported in Table 2. On the other hand, whereas *c*log*P* values do not discriminate between diastereomers, IAM-HPLC retention values do.

Our results show that there is a broadly agreement between *c*log*P* and log*K_IAM/W_* data. The only exceptions being compounds **3** and the **4**/**7** enantiomer pair. In particular, compound **3** is the only compound that has a *c*log*P* value lower than lacidipine and enantiomers **4**/**7** are the only compounds that have a log*K_IAM/W_* value lower than lacidipine. All other compounds have a lipohilicity higher than lacidipine and the other reference compounds and the enantiomers **5**/**6** are the most prone to partition in the immobilized membrane.

In order to better predict the ability of drugs to diffuse through the biological membranes, PAMPA has been used as a non cell based assay for measuring passive permeability of the investigated compounds [28]. Depending on the phospholipid type, PAMPA can mimic different adsorption/permeation environments. Lecithin in dodecane has been used for GI permeation studies (PAMPA-GI), whereas porcine polar brain lipid is used for BBB permeation assays (PAMPA-BBB) [29]. In brief our results indicate that all the synthesized compounds showed good permeability through GI membranes, being their permeability coefficient (Pe) higher than 2 × 10^−6^ cm/s. Moreover, they have also good brain permeability: in particular, enantiomers 4/7 seem very promising in BBB penetration, respect to the drug parent flurbiprofen (*Pe* ≥ 4×10^−6^ cm/s instead of *Pe* ≥ 2×10^−6^ cm/s) (Table 3) [30,31].

**Table 3:** PAMPA-GI and PAMPA-BBB permeability. *P_e_* (10^−6^ cm/s) of compounds **1**–**9** after 2 h of incubation for PAMPA-GI and after 18 h of incubation for PAMPA-BBB.

Time (h)	pH	Compounds								
		1	2	3	4–7	8–9	FLU	DAPT	LY-411575	LY-450139
2	7.4	4.8	4.1	2.0	4.6	2.6	2.1	2.6	2.5	1.8
	6.5	3.7	3.6	2.1	4.5	2.5	2.4	2.5	2.3	1.6
18	7.4	2.4	2.1	2.0	4.5	2.3	1.6	2.2	2.0	1.4

Compounds **1**–**9** were profiled for their *in vitro* toxicity and inhibitory activity on γ-secretase. For this purpose CTXTNA2 rat astrocytes have been treated with compounds **1**–**9** at concentrations ranging from 1 µM to 100 µM for 24 h, using LY-450139 and LY-411575 as the reference compounds [32]. With the exception of 1, 3 and 4, Aβ-42 production was reduced by increasing the concentration of investigated compounds without affecting the APP level (Table 4) and all compounds did not cause reduction in cell viability (data not shown) [33]. This behavior was consistent with that of LY-411575 and LY-450139 and was not related to compound toxicity because no reduction in cellular viability was observed (data not shown) [26].

**Table 4:** Expression of APP and Aβ-42 in CTXNA2 astrocytes exposed to different concentrations of the investigated **1**–**9** derivatives for 24 h (the most representative out of three different consistent experiments). Samples have been normalized by incubating membranes in the presence of β tubulin monoclonal antibody. Western blotting is the most representative out of three different consistent experiments.

	**C**		**1**		
			**1 µM**	**10 µM**	**100 µM**
**APP**	(63.1 ± 3.2) × 10^−2^		(93.1 ± 3.8) × 10^−2^	(89.4 ± 3.2) × 10^−2^	(120 ± 3.2) × 10^−2^
**Aβ-42**	(23.2 ± 0.8)×10^−2^		(29.2 ± 1.2) × 10^−2^	(43.5 ± 1.6) × 10^−2^	(45.8. ± 1.3) × 10^−2^
**2**			**3**		
**1 µM**	**10 µM**	**100 µM**	**1 µM**	**10 µM**	**100 µM**
(93.1 ± 2.6) × 10^−2^	(93.3 ± 3.5) × 10^−2^	(63.6 ± 2.8) × 10^−2^	(64.1 ± 2.3) × 10^−2^	(72.2 ± 2.1) × 10^−2^	(105 ± 3.2) × 10^−2^
(20.0 ± 1.1) × 10^−2^	(12.1 ± 0.6) × 10^−2^	(8.4 ± 0.2) × 10^−2^	(8.8. ± 0.4) × 10^−2^	(10.5 ± 0.4) × 10^−2^	(45.8. ± 1.3) × 10^−2^
**4**			**5**		
**1 µM**	**10 µM**	**100 µM**	**1 µM**	**10 µM**	**100 µM**
(51.3 ± 2.6) × 10^−2^	(39.9 ± 1.6) × 10^−2^	(60.1 ± 2.7) × 10^−2^	(41.1 ± 1.7) × 10^−2^	(44.2 ± 2.1) × 10^−2^	(49.2 ± 3.2) × 10^−2^
(11.0 ± 0.4) × 10^−2^	(12.1 ± 0.4) × 10^−2^	(16.2 ± 0.7) × 10^−2^	(17.4. ± 0.3) × 10^−2^	(17.1 ± 0.6) × 10^−2^	(17.4. ± 0.5) × 10^−2^
**6**			**7**		
**1 µM**	**10 µM**	**100 µM**	**1 µM**	**10 µM**	**100 µM**
(11.3 ± 0.4) × 10^−2^	(10.4 ± 0.2) × 10^−2^	(13.5 ± 0.5) × 10^−2^	(9.0 ± 0.3) × 10^−2^	(8.0 ± 0.3) × 10^−2^	(10.1 ± 3.2) × 10^−2^
(19.0 ± 0.6) × 10^−2^	(16.2 ± 0.5) × 10^−2^	(13.4 ± 0.7) × 10^−2^	(12.4. ± 0.1) × 10^−2^	(11.1 ± 0.3) × 10^−2^	(9.2 ± 0.3) × 10^−2^
**8**			**9**		
**1 µM**	**10 µM**	**100 µM**	**1 µM**	**10 µM**	**100 µM**
(82.3 ± 3.2) × 10^−2^	(68.1 ± 3.2) × 10^−2^	(94.4 ± 4.2) × 10^−2^	(68.7 ± 2.2) × 10^−2^	(69.2 ± 1.4) × 10^−2^	(94.3 ± 3.2) × 10^−2^
(31.1 ± 0.9) × 10^−2^	(25.0 ± 1.0) × 10^−2^	(35.2 ± 1.1) × 10^−2^	(43.2 ± 2.1) × 10^−2^	(38.4 ± 1.0) × 10^−2^	(14.0 ± 0.5) × 10^−2^
**LY411575**			**LY450139**		
**1 µM**	**10 µM**	**100 µM**	**1 µM**	**10 µM**	**100 µM**
(64.0 ± 2.4) × 10^−2^	(54.7 ± 2.0) × 10^−2^	(57.2 ± 1.2) × 10^−2^	(65.9 ± 2.3) × 10^−2^	(60.0 ± 1.6) × 10^−2^	(58.8 ± 2.5) × 10^−2^
(8.2 ± 0.1) × 10^−2^	(6.0 ± 0.1) × 10^−2^	(4.1 ± 0.2) × 10^−2^	(9.4. ± 0.3) × 10^−2^	(5.1 ± 0.1) × 10^−2^	(5.4 ± 0.2) × 10^−2^
**R-Flurbiprofen**			**S-Flurbiprofen**		
**1 µM**	**10 µM**	**100 µM**	**1 µM**	**10 µM**	**100 µM**
(60.0 ± 1.3) × 10^−2^	(54.5 ± 2.3) × 10^−2^	(52.1 ± 2.1) × 10^−2^	(60.6 ± 2.5) × 10^−2^	(65.2 ± 1.8) × 10^−2^	(53.2 ± 1.9) × 10^−2^
(20.5 ± 0.7) × 10^−2^	(19.1 ± 0.5) × 10^−2^	(14.1 ± 0.7) × 10^−2^	(18.8. ± 0.6) × 10^−2^	(19.0 ± 0.7) × 10^−2^	(15.4 ± 0.3) × 10^−2^
**DAPT**					
**1 µM**		**10 µM**		**100 µM**	
(65.4 ± 2.8) × 10−2		(62.1 ± 2.8) × 10−2		(58.3 ± 1.9) × 10−2	
(8.3 ± 0.2) × 10−2		(6.4 ± 0.1) × 10−2		(5.5 ± 0.3) × 10−2	

It is worth noting that enantiomers display different activity in agreement with the previously highlighted different activity of the (*R*)- and (*S*)-enantiomers of FLU on both COX-1 and Aβ42 [11]. It is particularly intriguing that the highly effective 2 and 7 compounds derive from the less studied (*S*)-enantiomer of FLU.

## 3. Experimental

### 3.1. General

All reagents were purchased from Sigma-Aldrich (St. Louis, MO, USA). LY-411575 and LY-450139 were bought from Haoyuan Chemexpress (Shanghai, China), whereas compounds **1**–**9** were synthesized in our labs. Microanalyses were performed on a 1106 Carlo Erba CHN analyzer, and the results were within (0.4%) of the calculated values. ^1^H- and ^13^C-NMR spectra were recorded on a Varian VXR 300-MHz spectrometer. Chemical shifts are reported in parts per million (δ) downfield from the internal standard tetramethylsilane (Me_4_Si). Optical rotations were taken at 20 °C with a Perkin/Elmer 241 polarimeter. Melting points were determined on a Büchi B-450 apparatus and are uncorrected. The LC-MS/MS system used consisted of an LCQ ion trap mass spectrometer (Thermo Finnigan, San Jose, CA, USA) equipped with an electrospray ionization (ESI) source. The capillary temperature was set at 300 °C and the spray voltage at 4.25 kV. The fluid was nebulized using nitrogen (N_2_) as both the sheath gas and the auxiliary gas. The identity of all new compounds was confirmed by elemental analysis, NMR data and LC-MS/MS system; homogeneity was confirmed by TLC on silica gel Merck 60 F254. Solutions were routinely dried over anhydrous sodium sulphate prior to evaporation. Chromatographic purifications were performed by Merck 60 70-230 mesh ASTM silica gel column. The HPLC system for solubility determination was a Waters Pump 600 Controller, equipped with a Waters 2996 photodiode array detector, a 20-μl Rheodyne injector and a Waters Symmetry RP-C18 column (4.6 mm × 150 mm, 5 µm); the mobile phase was a mixture of water/methanol 10/90. The flow rate was 1 mL/min and the wavelength for compound detection was 246 nm.

*(2-Fluoro-1,1'-biphenyl-4-yl)methanol* (**17**): NaBH_4_ (0.15 g, 4.0 mmol) was added to 2-fluoro-1, 1*'*-biphenyl-4-carbaldehyde (0.20 g, 1 mmol) in MeOH/THF (1:1, v/v, 4 mL). After 12 h at r.t., HCl 2N (1 mL) was carefully added and the product was extracted with Et_2_O. The organic layer was washed with brine, dried and evaporated. Crystallization from hexane afforded 17; Rf: 0.51 (CHCl_3_/acetone 7:3); yield 85%; ^1^H-NMR (CDCl_3_): δ 7.59–7.18 (m, 8H, ArH), 4.75 (s, 2H, CH_2_), 1.84 (bs, 1H, OH). 

*4-(Bromomethyl)-2-fluoro-1,1'-biphenyl* (**18**): The alcohol **17** (0.17 g, 0.84 mmol) was refluxed with 48% hydrogen bromide (1 mL) for 4 h at 120 °C. After cooling, water was added and the product was extracted with ether. The organic phase was purified by column chromatography with cyclohexane/Et_2_O 95:5 as eluent; Rf: 0.46 (cyclohexane/Et_2_O 95:5); yield 86%; ^1^H-NMR (CDCl_3_): δ 7.56–7.19 (m, 8H, ArH), 4.50 (s, 2H, CH_2_).

*(2-Fluoro-1,1′-biphenyl-4-yl)acetonitrile* (**19**): To a solution of KCN (0.11 g, 1.72 mmol) in water (2.5 mL) was slowly added a solution of **18** (0.46 g, 1.72 mmol) in ethanol (5 mL). After 2 h at 120 °C, the ethanol was evaporated and the product was extracted with ether and purified by column chromatography with cyclohexane/EtOAc 6:4; Rf: 0.56 (cyclohexane/EtOAc 6:4); yield 50%; ^1^H-NMR (CDCl_3_): δ 7.56–7.14 (m, 8H, ArH), 3.79 (s, 2H, CH_2_).

*(2-Fluoro-1,1'-biphenyl-4-yl)acetic acid* (**10**): To a solution of **19** (0.17 g, 0.83 mmol) in methanol (1.2 mL), 35% NaOH (4.8 mL) was added. The mixture was stirred for 8 h at 100 °C and then was cooled at r.t.. After acidification with HCl 2N, the precipitate formed was filtered, washed with water and dried under vacuum; Rf: 0.31 (EtOAc/Cyclohexane 6:4); yield 71%; ^1^H-NMR (CDCl_3_): δ 7.55–7.10 (m, 8H, ArH), 3.70 (s, 2H, CH_2_).

*N-Methylbiphenyl-2-amine* (**22**): To a stirred solution of biphenyl-2-amine (0.029 mol) in 2-propanol (125 mL) a solution 37% of formaldehyde in water (0.059 mol) was added and the solution was stirred for 1 h. In another flask, to a suspension of palladium on activated charcoal (0.003 mol) in 2-propanol (125 mL), a solution of ammonium formate (0.29 mol) in water (50 mL) was added. The two solutions were combined and the new suspension was stirred overnight at r.t. and then refluxed for 1 h. After cooling, the suspension was filtered over a Celite pad and the solvent was evaporated. The residue was dissolved in dichloromethane and washed with water. The organic layer was dried over Na_2_SO_4_ and evaporated. The crude product was purified by flash column chromatography *n*-hexane/EtOAc 98:2; Rf: 0.41 (*n*-hexane/EtOAc 98:2); yield: 43%; ^1^H-NMR (CDCl_3_): δ 7.50–7.42 (m, 4H, ArH); 7.38–7.35 (m, 1H, ArH); 7.24–7.22 (m, 1H, ArH); 7.14–7.10 (m, 1H, ArH); 6.84–6.80 (m, 1H, ArH); 6.73–6.70 (m, 1H, ArH), 4.05 (brs, 1H, NH); 2.78 (s, 3H, CH_3_).

*N-(Biphenyl-2-yl)-2-chloro-N-methylacetamide* (**23**): In a perfectly dried two-necked flask, to a cooled (ice bath) solution of **22** in dry DCM (10 mL), *N,N*-diisopropylethylamine (1.5 eq.) and 2-chloroacetyl chloride (1.5 eq.) were added. The solution was allowed to raise r.t. and stirred under a nitrogen stream for 2 h and then HCl 0.5 N was added. The two phases were separated and the organic layer was dried over Na_2_SO_4_ and evaporated. The crude product was recrystallized from EtOH; Rf: 0.60 (*n*-hexane/EtOAc 98:2); yield: 83%. ^1^H-NMR (CDCl_3_): δ 7.46–7.22 (m, 9H, ArH); 3.90 (d, 1H, CH_2_, *J* = 12 Hz); 3.75 (d, 1H, CH_2_, *J* = 12 Hz); 3.10 (s, 3H, CH_3_). 

*N-Methyl-5H-dibenzo[b,d]azepin-6(7H)-one* (**24**): In a perfectly dried three-necked flask, aluminum chloride (4 eq.) was added portionwise to **23** under a nitrogen stream. The mixture was vigorously stirred and heated at 100 °C for 1 h and then the temperature was raised to 170 °C until gas evolution ceased. After removing the oil bath, ice was added and the mixture was vigorously stirred for additional 30 min, then HCl 2N was added. The aqueous layer was extracted exhaustively with chloroform. The combined organic phases were washed with brine, dried over Na_2_SO_4_ and evaporated. The crude product was recrystallized from EtOH; Rf: 0.55 (*n*-hexane/EtOAc 98:2); yield: 59%. ^1^H-NMR (CDCl_3_): δ 7.65–7.60 (m, 2H, ArH); 7.42–7.25 (m, 6 H, ArH); 3.59–3.55 (m, 1H, CH_2_); 3.45–3.42 (m, 1H, CH_2_); 3.35 (s, 3H, CH_3_).

*(E)-7-(Hydroxyimmino)-5-metil-5H-dibenzo[b,d]azepin-6(7H)-one* (**25**): In a perfectly dried two-necked flask, to a cooled (ice bath) solution of **24** in dry toluene potassium-bis(trimethylsilyl)-amide (0.5 M solution in toluene, 1.1 eq.) was added dropwise, under a nitrogen stream. The solution was stirred for 20 min, and isopentyl nitrite (1.1 eq.) was added dropwise. The solution was allowed to raise to r.t. and was stirred overnight. The solution was quenched with HCl 1N; the two phases were separated and the organic layer was washed with brine, dried over Na_2_SO_4_ and evaporated. The crude product was purified by flash column chromatography (*n*-hexane/EtOAc 75:25); Rf: 0.40 (*n*-hexane/EtOAc 75:25); yield: 36%. ^1^H-NMR (CDCl_3_): δ 7.71–7.28 (m, 8H, ArH); 3.42 (s, 3H, CH_3_).

*7-Amino-5-methyl-5H-dibenzo[b,d]azepin-6(7H)-one* (**20**): A solution of **25** (200 mg) in EtOH (10 mL) was placed in a Parr autoclave. Palladium on activated charcoal (0.1 eq.) and HCl 2N were added. The suspension was hydrogenated at exactly 5 Bars for 24 h. The solution was filtered through a Celite pad and the solvent evaporated. The residue was added of HCl 4N and washed with EtOAc. The aqueous layer was basified with NaOH 2N and extracted exhaustively with EtOAc. The combined organic layers were dried over Na_2_SO_4_ and evaporated. No further purification was required; Rf: 0.20 (*n*-hexane/EtOAc 98:2); yield: 41%. ^1^H-NMR (CDCl_3_): δ 7.60–7.23 (m, 8H, ArH); 4.35 (s, 1H, CH); 3.36 (s, 3H, CH_3_); 2.38 (brs, 2H, NH_2_).

*Racemic resolution of the amine* **20**: The hydrochloride salt of **20** was suspended in dry diethyl ether and triethylamine (3 eq.) was added. To the mixture a solution of (1*S*)-(+)-menthylchloroformate (1.5 eq.) in dry diethyl ether was added dropwise. The suspension was stirred at r.t. for 2 h. The reaction was quenched with NaOH 2N, the phases were separated and the organic layer was dried over Na_2_SO_4_ and evaporated. The diastoisomers were separated by flash column chromatography (dichloromethane/cyclohexane 9:1) to afford compounds **21a** and **21b**. Overall yield: 89.7%. ^1^H-NMR (CDCl_3_) of compound **21a**: δ 7.58 (dd, 2H, ArH); 7.30-7.49 (m, 6H, ArH); 6.37 (d, 1H); 5.16 (d, 1H); 4.51 (m, 1H, CH); 3.37 (s, 3H, CH3); 2.09 (m, 1H); 1.93 (dd, 1H); 1.63 (m, 3H); 1.41 (m, 3H); 1.03 (m, 1H); 0.98 (d, 3H, CH3); 0.86 (d, 3H, CH3); 0.79 (d, 3H, CH3). ^1^H-NMR (CDCl_3_) of compound **21b**: δ 7.58 (dd, 2H, ArH); 7.30–7.49 (m, 6H, ArH); 6.37 (d, 1H); 5.16 (d, 1H); 4.51 (m, 1H, CH); 3.37 (s, 3H, CH_3_); 2.09 (m, 1H); 1.93 (dd, 1H); 1.63 (m, 3H); 1.41 (m, 3H); 1.03 (m, 1H); 0.88 (dd, 6H, 2CH_3_); 0.71 (d, 3H, CH_3_).

Compounds **21a** or **21b** (1 eq.) were dissolved in dry dichloromethane (10 mL) and the solution was cooled down (ice bath). Then trifluoroacetic acid (10 eq.) and triflic acid (3 eq.) were added dropwise. The reaction was stirred at 0 °C for 2 h and then was quenched with a saturated solution of NaHCO_3_. The two layers were separated and the aqueous phase was extracted with dichloromethane. The combined organic layers were dried over Na_2_SO_4_ and evaporated to afford one of the enantiomers of amine **20** as shown by TLC and its ^1^H-NMR spectrum. No further purification was required; Rf: 0.35 (*n*-hexane/EtOAc 98:2); Yield: 93%.

### 3.2. General Procedure for Synthesis of Compunds **11**–**13**

To a solution of flurbiprofen (0.82 mmol) in DCM (2.7 mL) was added a solution of DCC (0.17 g, 0.82 mmol) in DCM (2.7 mL). After 15 min, L*-*alanine-*t-*butylester hydrochloride (0.15 g, 0.82 mmol) in DCM (2.7 mL) and Et_3_N (107 µL, 0.82 mmol) was added. The mixture was stirred at room temperature for 3 h. The precipitated dicyclohexylurea was filtered and the solvent was evaporated. The product was purified by column chromatography with DCM/EtOAc 9:1 as eluent.

*tert-Butyl N-[(2R)-2-(2-fluoro-1,1′-biphenyl-4-yl)propanoyl]-L-alaninate* (**11**): Rf: 0.49 (DCM/EtOAc 9:1); yield 50%; ^1^H-NMR (CDCl_3_): δ 7.53-7.14 (m, 8H, ArH), 6.04 (d, 1H, *J* = 7.2 Hz, NH), 4.48–4.41 (m, 1H, αCH), 3.62–3.55 (m, 1H, CH), 1.53 (d, 3H, *J* = 7.2 Hz, CH_3_), 1.45 (s, 9H, CH_3_ *t*-Bu), 1.31 (d, 3H, *J* = 7.2 Hz, CH_3_).

*tert-Butyl N-[(2S)-2-(2-fluoro-1,1′-biphenyl-4-yl)propanoyl]-L-alaninate* (**12**): Rf: 0.49 (DCM/EtOAc 9:1); yield 50%; ^1^H-NMR (CDCl_3_): δ 7.58–7.19 (m, 8H, ArH), 6.07 (d, 1H, *J* = 7.2 Hz, NH), 4.46–4.41 (m, 1H, αCH), 3.62-3.55 (m, 1H, CH), 1.54 (d, 3H, *J* = 7.2 Hz, CH_3_), 1.41 (s, 9H, CH_3_ *t*-Bu), 1.36 (d, 3H, *J* = 7.2 Hz, CH_3_); ^13^C-NMR (DMSO-d_6_): δ 172.93 (s, 1C, CONH), 135.68–115.21 (10 s, 12 C, Ar), 82.33 (s, 1C, C *t*-Bu), 49.93 (s, 1C, αCH), 46.88 (s, 1C, CH), 28.41 (s, 3C, CH_3_ *t*-Bu), 19.41 (s, 1C, CH_3_), 18.10 (s, 1C, CH_3_).

*tert-Butyl N-[(2-fluoro-1,1′-biphenyl-4-yl)acetyl]-L-alaninate* (**13**): Rf: 0.38 (DCM/EtOAc 9:1); yield 74%; ^1^H-NMR (CDCl_3_): δ 7.56-7.09 (m, 8H, ArH), 6.14 (d, 1H, *J* = 6.6 Hz, NH), 4.50-4.46 (m, 1H, αCH), 3.60 (s, 2H, CH_2_), 1.44 (s, 9H, CH_3_ *t*-Bu), 1.36 (d, 3H, *J* = 7.2 Hz, CH_3_).

### 3.3. General Procedure for Synthesis of Compunds **14**–**16**

Compounds **11**–**13** (0.14 g, 0.38 mmol) were dissolved in TFA (7 mL). After 30 min at r.t., the solvent was evaporated the residue was repeatedly washed with ether, to give compounds **14**–**16** respectively in quantitative yield.

*N-[(2R)-2-(2-Fluoro-1,1′-biphenyl-4-yl)propanoyl]-L-alanine* (**14**): Rf: 0.28 (DCM/EtOAc 9:1); ^1^H-NMR (CDCl_3_): δ 8.58 (bs, 1H, COOH), 7.54–7.10 (m, 8H, ArH), 6.08 (d, 1H, *J* = 6.9 Hz, NH), 4.62–4.53 (m, 1H, αCH), 3.67–3.60 (m, 1H, CH), 1.54 (d, 3H, *J* = 7.2 Hz, CH_3_), 1.40 (d, 3H, *J* = 7.2 Hz, CH_3_).

*N-[(2S)-2-(2-Fluoro-1,1′-biphenyl-4-yl)propanoyl]-L-alanine* (**15**): Rf: 0.28 (DCM/EtOAc 9:1); ^1^H-NMR (CDCl_3_): δ 9.88 (bs, 1H, COOH), 7.54–7.09 (m, 8H, ArH), 6.10 (d, 1H, *J* = 6.9 Hz, NH), 4.58–4.53 (m, 1H, αCH), 3.64-3.61 (m, 1H, CH), 1.53 (d, 3H, *J* = 7.2 Hz, CH_3_), 1.42 (d, 3H, *J* = 7.2 Hz, CH_3_).

*N-[(2-Fluoro-1,1′-biphenyl-4-yl)acetyl]-L-alanine* (**16**): Rf: 0.18 (DCM/EtOAc 9:1); ^1^H-NMR (DMSO): δ 12.51 (bs, 1H, COOH), 8.48 (d, 1H, *J* = 7.5 Hz, NH), 7.53–7.15 (m, 8H, ArH), 4.20–4.16 (m, 1H, αCH), 3.51 (s, 2H, CH_2_), 1.27 (d, 3H, *J* = 7.5 Hz, CH_3_).

### 3.4. General Procedure for Synthesis of Final Products **1**–**3**

To a solution of compounds **14**–**16** (0.63 mmol) in DCM (2.1 mL) was added a solution of DCC (0.13 g, 0.63 mmol) in DCM (2.1 mL). After 15 min, L*-*phenylglycine-*t*-butylester hydrochloride (0.15 g, 0.63 mmol) in DCM (2.1 mL) and Et_3_N (82 µL, 0.63 mmol) was added. The mixture was stirred at room temperature for 2 h. The precipitated dicyclohexylurea was filtered and the solvent was evaporated. The final products were purified by column chromatography with DCM/EtOAc as eluent.

*tert-Butyl (2S)-({N-[(2R)-2-(2-fluoro-1,1'-biphenyl-4-yl)propanoyl]-L-alanyl}amino)(phenyl)acetate* (**1**): Rf: 0.59 (DCM/EtOAc 7:3); yield 70%; M.p: 105–107 °C; 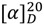 = + 127.2 (c = 1, EtOH); ^1^H-NMR (CDCl_3_): δ 7.54–7.08 (m, 14H, ArH and NH), 6.18 (d, 1H, *J* = 7.2 Hz, NH), 5.38 (d, 1H, *J* = 7.2 Hz, αCH), 4.61–4.52 (m, 1H, αCH), 3.55-3.48 (m, 1H, CH), 1.46 (d, 3H, *J* = 7.2 Hz, CH_3_), 1.38 (s, 9H, CH_3_ *t*-Bu), 1.34 (d, 3H, *J* = 6.9 Hz, CH_3_); ^13^C-NMR (CDCl_3_): δ 173.5 (s, 1C, CONH), 171.4 (s, 1C, CONH), 169.6 (s, 1C, COO), 160.0 (d, 1C, *J* = 987.6 Hz, CF), 142.6 (d, 1C, *J* = 29.4 Hz, Ar), 137.0 (s, 1C, Ar), 135.6 (s, 1C, Ar), 131.3 (d, 1C, *J* = 14.7 Hz, Ar), 129.2–127.2 (7s, 11C, Ar), 123.7 (d, 1C, *J* = 13.5 Hz, Ar), 115.4 (d, 1C, *J* = 94.5 Hz, Ar), 83.0 (s, 1C, C *t*-Bu), 57.3 (s, 1C, αCH), 48.9 (s, 1C, αCH), 46.6 (s, 1C, CH), 28.0 (s, 3C, CH_3_ *t*-Bu), 18.8 (s, 1C, CH_3_), 18.4 (s, 1C, CH_3_); MS (ESI) *m/z* 505 (M-H)^+^.

*tert-Butyl(2S)-({N-[(2S)-2-(2-fluoro-1,1'-biphenyl-4-yl)propanoyl]-L-alanyl}amino)(phenyl)acetate* (**2**): Rf: 0.59 (DCM/EtOAc 7:3); yield 40%; M.p: 106–108 °C; 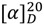 = + 213.4 (c = 1, EtOH); ^1^H-NMR (CDCl_3_): δ 7.54-7.10 (m, 14H, ArH and NH), 6.41 (d, 1H, *J* = 7.8 Hz, NH), 5.41 (d, 1H, *J* = 7.2 Hz, αCH), 4.64-4.59 (m, 1H, αCH), 3.65-3.58 (m, 1H, CH), 1.54 (d, 3H, *J* = 7.2 Hz, CH_3_), 1.38 (s, 9H, CH_3_ *t*-Bu), 1.26 (d, 3H, *J* = 6.6 Hz, CH_3_); ^13^C-NMR (CDCl_3_): δ 173.7 (s, 1C, CONH), 171.7 (s, 1C, CONH), 169.7 (s, 1C, COO), 160.0 (d, 1C, *J* = 987.6 Hz, CF), 142.7 (d, 1C, *J* = 30.9 Hz, Ar), 137.1 (s, 1C, Ar), 135.7 (s, 1C, Ar), 131.2 (d, 1C, *J* = 15.9 Hz, Ar), 129.3-127.3 (7s, 11C, Ar), 123.8 (d, 1C, J = 13.5 Hz, Ar), 115.4 (d, 1C, *J* = 94.2 Hz, Ar), 82.9 (s, 1C, C *t*-Bu), 57.2 (s, 1C, αCH), 48.9 (s, 1C, αCH), 46.6 (s, 1C, CH), 28.0 (s, 3C, CH_3_ *t*-Bu), 18.8 (s, 1C, CH_3_), 18.4 (s, 1C, CH_3_); MS (ESI) *m/z* 505 (M-H)^+^.

*tert-butyl (2S)-({N-[(2-fluoro-1,1'-biphenyl-4-yl)acetyl]-L-alanyl}amino)(phenyl)acetate* (**3**): Rf: 0.62 (DCM/EtOAc 9:1); yield 66%; M.p: 104–106 °C; 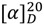 = + 170.3 (c = 1, EtOH); ^1^H-NMR (CDCl_3_): δ 7.53–7.09 (m, 14H, ArH and NH), 6.39 (d, 1H, *J* = 7.2 Hz, NH), 5.37 (d, 1H, *J* = 7.2 Hz, αCH), 4.62–4.58 (m, 1H, αCH), 3.60 (s, 2H, CH_2_), 1.36 (s, 9H, CH_3_ *t*-Bu), 1.30 (d, 3H, *J* = 7.2 Hz, CH_3_); ^13^C-NMR (CDCl_3_): δ 171.5 (s, 1C, CONH), 170.5 (s, 1C, CONH), 169.7 (s, 1C, COO), 160.0 (d, 1C, *J* = 990.0 Hz, CF), 137.0 (s, 1C, Ar), 136.0 (d, 1C, *J* = 33.0 Hz, Ar), 135.6 (s, 1C, Ar), 131.3 (d, 1C, *J* = 15.9 Hz, Ar), 129.2-127.1 (7s, 11C, Ar), 125.6 (d, 1C, *J* = 13.5 Hz, Ar), 117.8 (d, 1C, *J* = 93.3 Hz, Ar), 83.0 (s, 1C, C *t*-Bu), 57.3 (s, 1C, αCH), 48.9 (s, 1C, αCH), 43.0 (s, 1C, CH_2_), 27.9 (s, 3C, CH_3_ *t*-Bu), 18.3 (s, 1C, CH_3_); MS (ESI) *m/z* 491 (M-H)^+^.

### 3.5. General Procedure for Synthesis of Final Products **4**–**9**

The corresponding carboxylic acid (1 eq.) was dissolved in dry THF. **20a** or **20b** (1 eq.), *N*-(3-dimethylaminopropyl)-*N′*-ethylcarbodiimide hydrochloride (1 eq.), HOBt (1 eq.) and diisopropylethylamine (1 eq.) were added and the mixture was stirred at r.t. for 16 h. The reaction was quenched with HCl 0.5 N and the aqueous layer was extracted with DCM. The combined organic phases were washed twice with NaHCO_3_ 2.5% and once with brine. The organic layer was dried over Na_2_SO_4_ and evaporated.

*(2R)-2-(2-Fluoro-1,1'-biphenyl-4-yl)-N-[(7R)-5-methyl-6-oxo-6,7-dihydro-5H-dibenzo[b,d]azepin-7-yl]propanamide* (**4**): Rf: 0.63 (DCM/EtOAc 7:3); Yield: 44%; 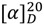 = +43.1 (c = 1, EtOH); M.p: 100–103 °C; ^1^H-NMR (CDCl_3_): δ 7.65–7.20 (m, 16H, ArH); 6.98–6.95 (m, 1H, NH); 5.41-5.37 (m, 1H, CH); 3.88–3.83 (m, 1H, CH); 3,38 (s, 3H, CH_3_); 1,59 (s, 3H, CH_3_); ^13^C-NMR (CDCl_3_): δ 171.4 (s, 1C, CONH), 169.2 (s, 1C, CONH), 162.7-111.4 (22s, 24C, Ar), 60.3 (s, 1C, αCH), 47.9 (s, 1C, αCH), 32.1 (s, 1C, CH_3_), 17.8 (s, 1C, CH_3_); MS (ESI) *m/z* 465 (M−H)^+^.

*(2R)-2-(2-Fluoro-1,1'-biphenyl-4-yl)-N-[(7S)-5-methyl-6-oxo-6,7-dihydro-5H-dibenzo[b,d]azepin-7-yl]propanamide* (**5**): Rf: 0.63 (DCM/EtOAc 7:3); Yield: 44%; M.p: 102–103 °C; 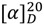 = +42.8 (c = 1, EtOH); ^1^H-NMR (CDCl_3_): δ 7.62-7.20 (m, 16H, ArH and 1H, NH); 5.35–5.32 (m, 1H, CH); 3.89–3.85 (m, 1H, CH); 3.35 (s, 3H, CH_3_); 1.62 (s, 3H, CH_3_); ^13^C-NMR (CDCl_3_): δ 172.2 (s, 1C, CONH), 170.1 (s, 1C, CONH), 163.2-111.2 (22s, 24C, Ar), 59.9 (s, 1C, αCH), 47.00 (s, 1C, αCH), 32.8 (s, 1C, CH_3_), 17.9 (s, 1C, CH_3_); MS (ESI) *m/z* 465 (M−H)^+^.

*(2S)-2-(2-Fluoro-1,1'-biphenyl-4-yl)-N-[(7R)-5-methyl-6-oxo-6,7-dihydro-5H-dibenzo[b,d]azepin-7-yl]propanamide* (**6**): Rf: 0.63 (DCM/EtOAc 7:3); Yield: 47%; M.p: 104–105 °C; 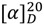 = −42.8 (c = 1, EtOH); ^1^H-NMR (CDCl_3_): δ 7.65–7.20 (m, 16H, ArH); 6.99-6.95 (m,1H, NH); 5.40-5.37 (m, 1H, CH); 3.88–3.83 (m, 1H, CH); 3.38 (s, 3H, CH_3_); 1.59 (d, 3H, CH_3_); ^13^C-NMR (CDCl_3_): δ 172.2 (s, 1C, CONH), 171.0 (s, 1C, CONH), 164.0-112.0 (22s, 24C, Ar), 61.0 (s, 1C, αCH), 48.2 (s, 1C, αCH), 32.4 (s, 1C, CH_3_), 18.0 (s, 1C, CH_3_); MS (ESI) *m/z* 465 (M-H)^+^.

*(2S)-2-(2-Fluoro-1,1'-biphenyl-4-yl)-N-[(7S)-5-methyl-6-oxo-6,7-dihydro-5H-dibenzo[b,d]azepin-7-yl]propanamide* (**7**): Rf: 0.63 (DCM/EtOAc 7:3); Yield: 47%; M.p: 102–104 °C; 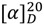 = −43.1 (c = 1, EtOH); ^1^H-NMR (CDCl_3_): δ 7.62–7.20 (m, 16H, ArH and 1H, NH); 5.36–5.32 (m, 1H, CH); 3.90–3.85 (m, 1H, CH); 3.35 (s, 3H, CH_3_); 1.62 (s, 3H, CH3); ^13^C-NMR (CDCl_3_): δ 172.1 (s, 1C, CONH), 170.2 (s, 1C, CONH), 164.8–112.3 (22s, 24C, Ar), 60.4 (s, 1C, αCH), 48.5 (s, 1C, αCH), 32.6 (s, 1C, CH_3_), 17.6 (s, 1C, CH_3_); MS (ESI) *m/z* 465 (M-H)^+^.

*2-(2-Fluoro-1,1'-biphenyl-4-yl)-N-[(7R)-5-methyl-6-oxo-6,7-dihydro-5H-dibenzo[b,d]azepin-7-yl]-acetamide* (**8**): Rf: 0.56 (DCM/EtOAc 7:3); Yield: 55%; M.p: 96–100 °C; 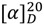 = +0.2 (c = 1, EtOH); ^1^H-NMR (CDCl_3_): δ 7.62-7.16 (m, 17H, ArH and NH); 5.36-5.31 (m, 1H, CH); 3.77 (s, 2H, CH_2_); 3.36 (s, 3H, CH_3_); ^13^C-NMR (CDCl_3_): δ 172.2 (s, 1C, CONH), 171.0 (s, 1C, CONH), 164.0–112.0 (22s, 24C, Ar), 61.0 (s, 1C, αCH), 45.2 (s, 1C, CH_2_), 32.4 (s, 1C, CH_3_); MS (ESI) *m/z* 451 (M−H)^+^.

*2-(2-Fluoro-1,1'-biphenyl-4-yl)-N-[(7S)-5-methyl-6-oxo-6,7-dihydro-5H-dibenzo[b,d]azepin-7-yl]-acetamide* (**9**): Rf: 0.56 (DCM/EtOAc 7:3); Yield: 79%; M.p: 96–100 °C; 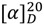 = −0.2 (c = 1, EtOH); ^1^H-NMR (CDCl_3_): δ 7.62-7.16 (m, 17H, ArH and NH); 5.35–5.31 (m, 1H, CH); 3.77 (s, 2H, CH_2_); 3.36 (s, 3H, CH_3_). ^13^C-NMR (CDCl_3_): δ 171.4 (s, 1C, CONH), 169.2 (s, 1C, CONH), 162.7–111.4 (22s, 24C, Ar), 60.3 (s, 1C, αCH), 46.2 (s, 1C, CH_2_), 32.1 (s, 1C, CH_3_); MS (ESI) *m/z* 451 (M−H)^+^.

### 3.6. Water Solubility

Compounds **1**–**9** (50 mg) were placed in deionized water (1 mL), shaken at 25 °C for 1 h to ensure the solubility equilibrium and then centrifuged. The supernatant (20 µL) was analyzed by HPLC [18].

### 3.7. FaSSIF Solubility

The Fasted State Simulated Intestinal Fluid (FaSSIF) medium was prepared as shown in Table 5 [34].

**Table 5:** FaSSIF composition.

pH	6.8
KH_2_PO_4_ (mM)	29
Bile salts (mM)	2.5
Phospholipids (mM)	0.5

An excess of each compound (about 2 mg) was added to a screw cap vial containing 1 mL medium heated to 37 °C. The sample was gently stirred for 24 h and then it was centrifuged at 4,500 rpm for 15 min: all these procedures were conducted at 37 °C. The supernatant was filtered, diluted with a same volume of methanol and analyzed by HPLC.

### 3.8. Lipophilicity

*n*-Octanol/water partition coefficients were theoretically calculated by the program *c*log*P* for windows version 2.0 (Biobyte Corp., Claremont, CA, USA). Affinity for membrane phospholipids, so called “phospholipophilicity”, was measured by HPLC on a phospholipid stationary phase. A 600E liquid chromatograph (Waters-Millipore, Milford, MA, USA), equipped with a 7125 Rheodyne injection valve and a 486 UV detector (Waters) set at 246 nm, was used. The column was an IAM.PC.MG (Immobilized Artificial Membrane-PhosphatidylCholine-MethylGlycolate) (4.6 mm × 150 mm) (Regis Chemical Company, Morton Grove, IL, USA). The mobile phase was 0.01 M phosphate buffer at pH 7.0 and acetonitrile at percentages ranging from 10 to 35 percent (v/v) (from 1.0 to 4.0 mL/min). The chromatography was carried out at room temperature. All samples were dissolved in methanol (*ca.* 10^−3^ M) and 20 μL samples were injected in the chromatograph. Chromatographic retention data are expressed as the logarithm of the capacity factor, log k, deﬁned as:

log k = log [(t_r_ − t_0_)/t_0_] (1)

where t_r_ and t_0_ are the retention times of the drug and a non-retained compound (citric acid), respectively. The log k values relative to 100% aqueous eluent (logK_IAM/W_) were calculated by performing a polycratic method of extrapolation [35,36]. Each compound was eluted with at least three different mobile phases containing acetonitrile in different percentages (φ). Linear relationships between log k and φ values were found for all the compounds in the range of eluent composition examined (r^2^ ≥ 0.994). All values of log k reported are the average of at least three measurements; the 95% conﬁdence interval never exceeded 0.04 for each log k value. Possible occurrence of retention changes due to column aging was monitored by checking the retention times of ﬁve test compounds (amlodipine, p-nitroaniline, toluene, isradipine, and ketoprofen). During the study no retention value of test compounds changed more than 4% and no correction was done to the retention values experimentally determined for the analytes.

### 3.9. PAMPA Method

The following protocol was applied to measure the Pe through the artificial membrane to predict oral absorption and BBB permeation. In order to study the g.i. permeability, a 2% solution (w/v) of egg lecithin (phosphatidyl choline > 98%, PC) in dodecane was employed as an artificial membrane solution. The effective permeability of BBB was measured using phospholipid mixture from porcine polar brain lipid extract, composed by PC 12.6%, phosphatidylethanolamine (PE) 33.1%, phosphatidylserine (PS) 18.5%, phosphatidylinositol (PI) 4.1%, phospatidic acid (PA) 0.8% and 30.9% of other compounds (purchased from Avantis Polar Lipids, Alabaster, AL, USA). Each donor filtration plate well was then impregnated with 5 µL of this solution carefully, and immediately after 150 µL of phosphate buffer (pH 7.4/pH 6.5), containing co-drug 1 500 µM and iPrOH 20% as co-solvent was added. Then the drug-filled donor plate was placed into the acceptor plate that had been prefilled with the same buffer (300 µL) as acceptor solution. After plate lid was replaced, the resulting assembled donor-acceptor plates were incubated at r.t. for 2 h for PAMPA-GI and 18 h for PAMPA-BBB, following which drugs concentration in the acceptor and donor solutions were determined by HPLC [37].

Log Pe can be calculated from the equation below:

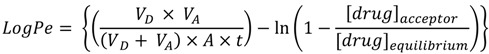
(2)
where Pe is the effective permeability coefficient (cm x s^−1^), V_D_ is volume of donor compartment (0.15 cm^3^) and V_A_ is volume of acceptor compartment (0.30 cm^3^), A is effective filter area (0.28 cm^2^), t is incubation time for the assay (s), C_acceptor_ is the concentration of the compound in the acceptor compartment at the completion of the assay, and C_equilibrium_ is the concentration of compound at theoretical equilibrium.

### 3.10. Cell Culture and Drug Treatment

CTXTNA2 cell line, established from primary cultures of type 1 astrocytes from brain frontal cortex tissue of 1 day old rats, was obtained by ECACC (Salisbury, UK). Cells have been maintained in DMEM, 10% FBS at 37 °C, 5% CO_2_ until sub-confluence and incubated for 24 h with a DMSO solution of drug (concentrations ranging from 1 µM to 100 µM). For detection of cell viability, a Trypan blue dye exclusion test has been performed [33].

### 3.11. Western Blotting

Cell lysates (20 µg) have been electrophoresed and transferred to nitrocellulose membrane. Nitrocellulose membranes, blocked in 5% non-fat milk, 10 mmol/L Tris pH 7.5, 100 mmol/L NaCl, 0.1% Tween-20, have been probed with mouse APP (Millipore, Billerica, MA, USA) and Aβ (Alpha Diagnostic Intl, San Antonio, TX, USA) monoclonal antibodies (Santa Cruz Biotechnology, Santa Cruz, CA, USA), then incubated in the presence of specific enzyme conjugated IgG horseradish peroxidase. Immunoreactive bands have been detected by ECL detection system (Amersham Intl., Buckinghamshire, UK) and analysed by densitometry. Densitometric values, expressed as Integrated Optical Intensity (I.O.I.), have been estimated in a CHEMIDOC XRS system by the QuantiOne 1-D analysis software (BioRad, Richmond, CA, USA). Values obtained have been normalized basing on densitometric values of internal β tubulin. Statistical analysis has been performed using the analysis of variance (ANOVA). Results have been expressed as mean ± SD. Values of *p* < 0.05 have been considered statistically significant.

### 3.12. MTT Assay

Viability of cells was assessed by measuring the formation of a formazan from MTT spectrophotometrically via commercial kits (Cayman Chemical^®^, Ann Arbor, Michigan, USA). At the end of the experiment, the cells were incubated with 0.7 mg/mL MTT for 30 min at 37 °C. After washing the blue formazan was extracted from cells with isopropanol/formic acid (95:5) and was photometrically determined at 560 nm. The density of formazan formed in untreated cells was taken as 100% viability.

## 4. Conclusions

In conclusion, the data obtained highlight that both a high *c*log*P* and a high log*K_IAM/W_* are necessary for the derivatives to be active. It appears also that, contrary to data on properties of FLU, the more *in vitro* active novel compounds are characterized by a permeability in PAMPA-GI much higher (about 20 times) than that in PAMPA-BBB. These results allow us to confirm that the newly synthesized compounds are not toxic and are able to reduce Aβ levels, especially compounds 2 and 7. However, the site of action of these compounds has not been identified yet. Nevertheless the similar stereochemistry of the latter effective compounds and the evidenced important role of lipophilicity and PAMPA permeability could help in elucidating this point. The improvement of potency and the investigation of *in vivo* activity are subject to further investigations. In particular studies on Aβ-40 and 42 quantification and Notch processing are under way to understand if compound 2 and 7 behave as GSIs or GSMs.

## Abbreviations

Aβ: β amyloid
AD: Alzheimer’s disease
APP: amyloid precursor protein
BBB: blood brain barrier
EDCI: *N*-(3-dimethylaminopropyl)-*N′*-ethylcarbodiimide hydrochloride
FASSIF: fasted state simulated intestinal fluid
FLU: flurbiprofen
GI: gastrointestinal
GSI: γ-secretase inhibitors
GSM: γ-secretase modulators
IAM: immobilized artificial membrane
KHMDS: potassium hexamethyldisilazide
NSAIDs: non-steroidal anti-inflammatory drugs
PAMPA: parallel artificial membrane permeability
SP: senile plaque
